# *Gracilaria edulis* extract induces apoptosis and inhibits tumor in Ehrlich Ascites tumor cells *in vivo*

**DOI:** 10.1186/1472-6882-13-331

**Published:** 2013-11-25

**Authors:** Satyajit Patra, Meenakshi Sundaram Muthuraman

**Affiliations:** 1 Medical University of the Americas, Charlestown, Nevis, West Indies; 2 Department of Biotechnology, School of Chemical and Biotechnology, SASTRA University Thanjavur, 613401, TamilNadu, India

**Keywords:** *Gracilaria edulis* J. Ag, Ehrlich ascites tumor, Antitumor, Apoptosis, Toxicology

## Abstract

**Background:**

Marine environment is inestimable for their chemical and biological diversity and therefore is an extraordinary resource for the discovery of new anticancer drugs. Recent development in elucidation of the mechanism and therapeutic action of natural products helped to evaluate for their potential activity.

**Methods:**

We evaluated *Gracilaria edulis* J. Ag (Brown algae), for its antitumor potential against the Ehrlich ascites tumor (EAT) *in vivo* and *in vitro*. Cytotoxicity evaluation of Ethanol Extract of *Gracilaria edulis* (EEGE) using EAT cells showed significant activity. *In vitro* studies indicated that EEGE cytotoxicity to EAT cells is mediated through its ability to produce reactive oxygen species (ROS) and therefore decreasing intracellular glutathione (GSH) levels may be attributed to oxidative stress.

**Results:**

Apoptotic parameters including Annexin-V positive cells, increased levels of DNA fragmentation and increased caspase-2, caspase-3 and caspase-9 activities indicated the mechanism might be by inducing apoptosis. Intraperitoneally administration of EEGE to EAT-bearing mice helped to increase the lifespan of the animals significantly inhibited tumor growth and increased survival of mice. Extensive hematology, biochemistry and histopathological analysis of liver and kidney indicated that daily doses of EEGE up to 300 mg/kg for 35 days are well tolerated and did not cause hematotoxicity nor renal or hepatotoxicity.

**Conclusion:**

Comprehensive antitumor analysis in animal model and in Ehrlich Ascites Tumor cells was done including biochemical, and pathological evaluations indicate antitumor activity of the extract and non toxic *in vivo*. It was evident that the mechanism explains the apoptotic activity of the algae extract.

## Background

All through the medical history, nature is the excellent and reliable source of new drugs, including anticancer agents. Natural sources like plants and marine products have always been useful sources of antitumor or cancer prevention compounds [1,2]. From the currently used anticancer chemotherapeutic drugs, approximately 70% are derived in from natural sources [3] including some drugs under clinical trials obtained from marine source [4,5]. Evidence from recent publication indicates that marine natural products, especially the secondary metabolites from marine organisms, are potential source and give high yield anticancer drugs than terrestrial sources [6,7]. In recent years compounds like Arc-C (Cytarabine, an antileukemic drug) and trabectedin (Yondelis, ET-743, an agent for treating soft tissue sarcoma) were developed from marine sources [8,9]. Fungi obtained from marine source are source of structurally unique and biologically active secondary metabolites [10]. Number of preclinical anticancer lead compounds obtained from marine-derived organism has been increasing rapidly in last few years [11-13]. In many cases the natural occurring compounds are more effective and do not have considerable undesired consequences compared with synthetic drugs [14]. Compounds from natural source are studied extensively with respect to structural modification in order to explore their further use in pharmacy and medicine in the prevention and treatment of cancer [15].

*Gracilaria edulis* (S.G. Gmelin) P.C. Silva, a major Indian agarophyte and an edible marine alga is commonly found in Indian coast [16]. In a previous study, we reported the role of *G. edulis* in improvement in survival and cancer treatment [17]. We continued further to establish the role of *G. edulis* as anticancer drug and in this study we did an extensive evaluation of the activity to understand the mechanism. Increase in life span in the Ehrlich ascites tumour (EAT) cells bearing mice after treatment with ethanolic extract of *Gracilaria edulis* (EEGE) and results from the biochemical parameters encouraged us to perform the detailed study for this novel anticancer drug.

## Methods

### Reagents

Culture medium RPMI 1640, fetal bovine serum (FBS), HEPES and L-glutamine were purchased from Life Technologies (Grand Island, NY, USA). Trypan blue, MTT were obtained from Sigma Aldrich (St. Louis, MO, USA). Annexin-V-fluorescein isothiocyanate (FITC) and propidium iodide (PI) were from BD Biosciences (San Jose, CA, USA), and 2,7-dichlorodihydrofluorescein diacetate (H2-DCFDA) was from Molecular Probes/Invitrogen (Eugene, OR, USA). Caspase-2, caspase-3 and caspase-9 activities were evaluated by using commercial available kits from R&D Systems (Minneapolis, MN, USA). For evaluation of hepatic enzymes such as aspartate amino transferase (AST), alanine amino transferase (ALT), alkaline phosphatase (ALP), and lactate dehydrogenase (LDH) commercial kits were used (Span Diagnostics Ltd., Vadodara, Gujarat, India).

### Collection and extraction of EEGE

Fresh algae of *G. edulis* were collected from the regional sea shore during the month of December in the Mandapam region, Tamil Nadu. Alcoholic extract of the algae was prepared as described earlier and the presence of biologically active components including alkaloids, flavonoids, sterols, terpenoids, proteins, saponins, phenols, coumarins, tannins and glycosides was documented using spectrophotometric analysis [17]. No specific permission was required for the collection of these algae as these were collected from regional sea shore, not covered by any regulatory body and private land. This study does not involve any endangered or protected species. A voucher specimen of this algae was matched with the local herbarium authentic specimen (Herbarium no. AC.3.1.1.5) housed at Central Marine Fischeries Research Institute, Cochin, Kerala, India and was deposited in the herbarium.

### Animals and mouse tumor model

Adult swiss albino mice weighing between 25–30 g were procured from Tamilnadu Veterinary and animal Science University, Chennai. The animals were kept in well-ventilated cages and fed with commercial food and water *ad libitum* and raised under specific pathogen-free conditions. The study was conducted with necessary ethical clearance from Institutional Animal Ethics Committee (IAEC) of Srimad Andavan Arts & Science College. EAT cells were provided as courtesy sample by Amala Cancer Research Center, Thrissur, India. Ascitic tumor cells were counted by trypan blue dye exclusion method and always found to be 95% or more viable. Cells were maintained in mice in ascites form by successive transplantation of 6×10^6^ cells/mouse in a volume of 0.2 ml in PBS [18].

### *In vitro* EAT cell culture

Following inoculation of EAT cells in mice abdominal cavity, after ten days the cells were collected by needle aspiration, washed in saline and erythrocytes were removed by washing in

35 mM NaCl. Cells were cultured in RPMI 1640 supplemented with HEPES (25 mM), L-glutamine (2 mM), sodium bicarbonate (25 mM), 10% FBS, 2-mercaptoethanol (50 μM) and antibiotics (100 U/ml penicillin and 100 μg/ml streptomycin) at 37°C in 5% of CO_2_ incubator. Viability and cell density were determined by the trypan blue dye exclusion test.

### Evaluation of EEGE cytotoxicity in EAT cells

In a 96 well plate, EAT cells (3×10^5^/ml) in RPMI 1640 with 10% FBS were seeded in quadruplicate. EEGE was dissolved in PBS which final concentration was adjusted to less than 0.1% (v/v) of the solvent in culture medium. The cells were treated with EEGE while control samples were treated with the corresponding volume of culture medium containing PBS. All samples were incubated in 5% CO_2_ incubator for 72 hours at 37°C in a 100% humidity atmosphere. Cell proliferation was determined using the standard MTT assay [19] and the phosphatase activity assay [20].

### Leukocyte culture and evaluation of EEGE cytotoxicity

Peripheral human blood was obtained from healthy adult volunteer with prior ethical approval and diluted with an equal volume of RPMI 1640 medium. Mononuclear cell was isolated using Ficoll-Hypaque density gradient separation solution, washed twice in RPMI1640 medium. Cells were suspended in RPMI1640 medium supplemented with 2 mM glutamine, antibiotics and 10% FBS. Leukocytes at a density of 1 × 10^6^ plating cells/ml were cultured with 5 μg/ml of phytohemagglutinin in 96-well microtiter plates. Cells were incubated with EEGE in a 5% CO_2_ incubator for 72 h at 37°C. Control samples were treated with the corresponding volume of culture medium containing less than 0.1% PBS. After treatment, cell proliferation was determined using the MTT reduction assay [19].

### Glutathione assay

EAT cells (5 × 10^6^) were treated with various concentrations of EEGE including 0, 25, 50 and 100 μg/ml for 72 hours were washed with PBS. Total and reduced glutathione concentration in the cells was estimated by Glutathione Assay Kit from Sigma. The cells were processed as per kit protocol. The sample is first deproteinized with the 5% 5-sulfosalicylic acid solution. Glutathione content of the sample is then assayed using a kinetic assay in which catalytic amounts of glutathione cause a continuous reduction of 5,5′-dithiobis-(2-nitrobenzoic) acid (DTNB) to TNB. The oxidized glutathione formed is recycled by glutathione reductase and NADPH. The product, TNB, is assayed colorimetrically at 412 nm.

### Reactive oxygen species (ROS) measurement

EAT cells (5 × 10^6^) were treated with EEGE (50 μg/ml) for 8, 12 and 24 hours in a 96-well plate followed by analysis of intracellular ROS using the oxidation-sensitive fluorescent probe 2,7-dichlorofluorescein diacetate (DCFH-DA). DCFH-DA enters cells and is hydrolyzed to membrane-impermeant dichlorofluorescein, which reacts with ROS to form the highly fluorescent dichlorofluorescein. Briefly, EAT cells were loaded with 5 μM DCFH-DA for the last 30 min of EEGE and the fluorescence of the generated DCF was measured in a fluorimeter plate reader at 490 nm excitation and 538 nm emission. Corrected values according to the cell number estimated by the trypan blue assay and the amount of ROS formed was expressed relative to the control [21,22].

### DNA fragmentation

DNA fragmentation was evaluated by using protocol described by McGahon et al. (1995) with modification. EAT cells (5 × 10^6^) were incubated with the EEGE at different concentrions (0, 25, 50, 100 μg/ml) for 48 hours to estimate the DNA fragmentation at 37°C. After 48 hours, cell suspension containing 4-6×10^5^ cells in a microcentrifuge tube was centrifuged for 5 min at 2000 × g, 4°C. The cell pellet was processed to isolate the DNA as per the protocol followed by addition of 10 pg/ml RNase (Boehringer Mannheim, Indianapolis, IN) and were incubated at 50°C for 1 hour. DNA was purified using DNA purification kit from Qiagen as per manufactures protocol. Extracted DNA was dissolved in 50 μL TE buffer, and electrophoresis was performed on a 1.8% agarose gel containing ethidium bromide [23] and densitometric analysis of bands was done by ImageJ Software (NIH, USA).

### Determination of caspases activities

EAT cells (5 × 10^6^) were incubated with EEGE (0, 25, 50, 100 μg/ml) for 72 hours and followed by measurement of caspase-2, caspase-3 and caspase-9 activities using colorimetric protease kits as per the manufacturer’s protocol. To prepare total cellular protein, cells were pelleted by centrifugation and lysed on ice and total protein concentration in the lysate was measured. With each X-pNA substrate (200 μM final concentration) 200 μg of proteins were incubated at 37°C for 4 hours in a 96 well plate. The absorbance of the samples was measured at 405 nm and the increase in the caspase activity of treated cells was determined by comparing the results with the untreated cells and standard drug after background correction.

### Annexin V-FITC/PI analysis

Detection of apoptosis was performed using the Annexin V-FITC/PI apoptosis detection kit according to manufacturer’s protocol. Briefly, both EEGE treated and untreated EAT cells were washed in 1× PBS and stained with annexin V-FITC conjugate and PI. Cells were then analyzed by flow cytometry (BD FACSCalibur, USA) using BD CellQuest acquisition and analysis software.

### Antitumor evaluation

The antitumor activity of EEGE was evaluated by measuring survival time and tumor growth inhibition. Mice were inoculated with 6×10^6^ EAT cells by i.p. route. After 24 h, EEGE was administered by i.p. injections of 0.2 ml per mouse. Endpoint of experiments was determined by spontaneous death of animals. The ascitic fluid from the peritoneal cavity of tumor bearing mice was quantitatively isolated by peritoneal lavage after death. The total number of tumor cells was counted by the trypan blue exclusion method. EEGE solutions were prepared in PBS containing 10% Tween 80. Control mice received the vehicle control as i.p. injection of 10% Tween 80 in PBS for the same time period.

### *In vivo* toxicological studies

An extended 35-day toxicity study of EEGE was conducted in adult swiss albino mice with daily doses of 300 mg/kg. Two groups of six animals each were used for toxicity study where animals had free access to water and food. The first group was served as vehicle control and second group was given 300 mg/kg of EEGE in PBS, containing 10% Tween 80, by i.p. injections of 0.2 ml per mouse, once daily. Every day morning clinical signs of gross toxicity, behavioral changes and mortality were observed. Body weight of individual animal was recorded before and at the end of experiment. After 24 hours of the administration of the last dose, the animals were euthanized by anesthetizing with ketamine hydrochloride and xylazine hydrochloride administered [24]. Whole blood was then sampled from the retro-orbital sinus with suitable hematological tube. For hematology assays 150 μl was retained and the remaining volume was used for serum biochemistry. The blood for serum biochemistry was allowed to clot at room temperature and was centrifuged at 3000 rpm for 10 min for serum separation. After blood collection, all mice were killed by cervical dislocation and liver and kidneys were collected, washed in PBS, fixed with 10% formalin and stored for histopathological examination.

### Hematological and biochemical analyses

Whole blood was immediately analyzed for complete blood count with differential and platelet count using the fully automated analyzer (Hitachi, Tokyo, Japan). Serum samples were analyzed for AST, ALT activities, and also ALP and LDH levels by commercial kits as per manufacturer’s instruction.

### Histopathological analysis

Routine histological processes were employed for paraffin inclusion, sectioning and H/E staining of liver and kidney from mice treated with EEGE and vehicle control. A histopathologist performed a complete examination of the tissues.

### Statistical analysis

All *in vitro* experiments were performed in triplicate and results are represented as means ± SD. Significant differences among groups was performed by ANOVA followed by Tukey test. The survival of mice was demonstrated using the Kaplan–Meier method and the logrank (Cox–Mantel) statistical test was applied to compare the curves for non-parametric procedures. Values where p < 0.05, differences were considered significant at representing two-sided test of statistical significance.

## Result

### Effect of EEGE on proliferation and viability of EAT cells and lymphocytes *in vitro*

**Figure 1:**
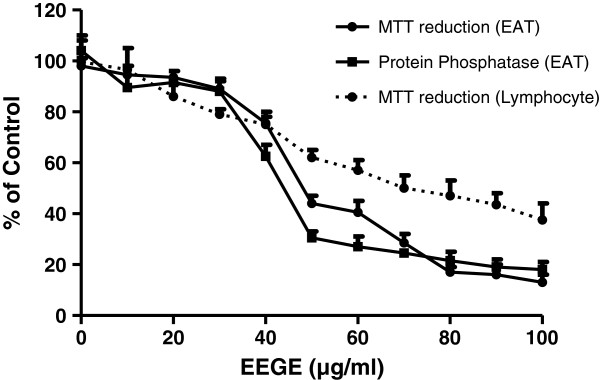
**Cytotoxicity of EEGE in EAT cells (3 × 10**^**5 **^**cells/ml; solid line) and human lymphocytes (1 × 10**^**6 **^**cells/ml; dashed line) after 72 hours of incubation.** Effects of EEGE on MTT reduction (▲) and protein phosphatase activity (●) is expressed relative to control cell viability (100%) and each point represents the mean ± S.D.

**Figure 2:**
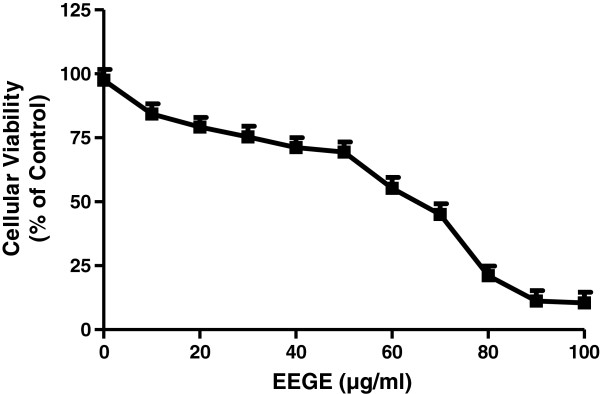
**Effect of EEGE on the viability of EAT cells (3 × 10**^**5 **^**cells/ml) determined by trypan blue exclusion test after 72 hours of incubation.** The results present the mean ± S.D. of three experiments run in quadruplicate.

Cytotoxicity induced by EEGE in EAT cells was evaluated by using MTT reduction and phosphatase activity with different concentrations of EEGE after 72 hours of treatment (Figure 1). EAT cells were exposed to various concentrations of EEGE and it resulted in a significant negative effect in cell proliferation, with the IC_50_ of 45 μg/ml observed in MTT reduction and phosphatase activity assays. At low concentrations of EEGE, a non-significant acceleration of cell growth was observed (Figure 1). By using trypan blue dye exclusion method, the effect of EEGE in EAT cells *in vitro* assay we also confirmed the above observation. Cells exposed to EEGE for 72 hours decreased cell viability in a dose-dependent manner (Figure 2). At 50 μg/ml dose the EAT cells viability was close to 65% and the maximum decrease of 15% was observed at 100 μg/ml. From these results, we were convinced that the EEGE potently inhibits the proliferation and viability of EAT cells and we continued with further investigations. EEGE was able to inhibit proliferation of human lymphocytes also, however the potency was not comparable to EAT cells, presenting IC_50_ nearly 1.5 fold higher as 70 μg/ml than for EAT cells, as observed in the MTT assay after 72 hours of incubation with EEGE in the same range of concentrations (Figure 1). For further *in vitro* analysis EEGE was used at 25, 50 and 100 μg/ml for cellular assays.

### Cellular glutathione and reactive oxygen species (ROS) levels altered by EEGE in EAT cells

**Figure 3:**
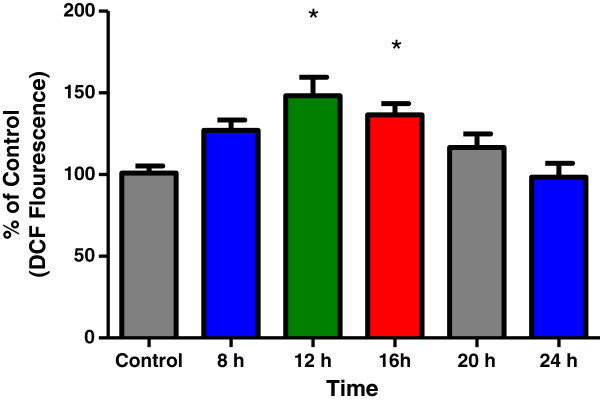
**Time-course effect of EEGE on ROS generation in EAT cells.** The fluorescence intensity of DCF was monitored at 538 nm, with excitation wavelength set at 490 nm, and used to indicate the level of intracellular peroxides formation. Changes in DCF fluorescence in tumor cells were measured at 8, 12, 16, 20 and 24 hours after treatment with 50 μg/ml of EEGE, *p < 0.01 compared to control and 24 h-time points (ANOVA, Tukey test). The results express the mean ± S.D. of three independent experiments run in duplicate.

ROS is known to be a key player in highly organized cellular functions such as pathways of signal transduction and apoptosis [25] and a role for oxidative signaling in the cytotoxicity of marine product in cancer cells has been previously reported [26]. In this context we investigated a potential role of oxidative stress in the alteration of cellular sensitivity to EEGE. EAT cells treated with EEGE for 30 min were used for estimation of ROS level after the addition of DCFH-DA. The time-course effect of EEGE on the EAT cell intracellular peroxide levels is presented in Figure 3. Intracellular ROS production was observed at 8–24 hours after incubation of tumor cells with 50 μg/ml of EEGE as compared to control cells, and found to be significantly increased (p < 0.01). Increase in peroxides amounts generated by EAT cells was also noted to be time-dependent, with significantly higher (p < 0.01) at the beginning of treatment such as 8 and 12 hours in comparison with the 24 hours time point and the peroxides levels reached to normal after 24 hours exposure in EAT cells.

**Figure 4:**
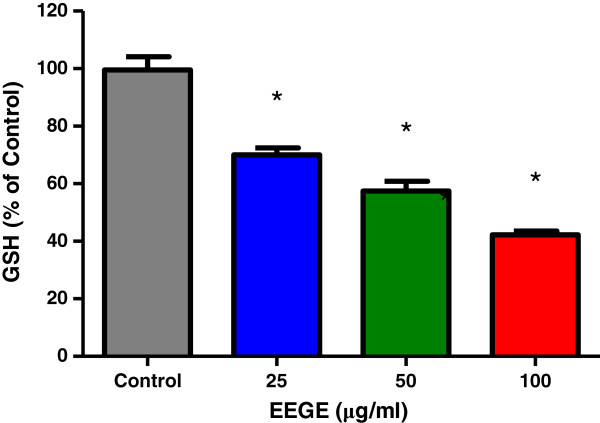
**Alteration on GSH levels of EAT cells (3 × 10**^**5 **^cells/ml) treated with EEGE for 72 h, *p < 0.01 compared to control cells (ANOVA, Tukey test). The results present the mean ± S.D. of three experiments run in duplicate.

With observation of an oxidative cytotoxic mechanism, we further measured the level of glutathione (GSH), the major non-protein thiol in mammalian cells with chemoprotective action, particularly associated with antioxidant defense. EAT cells treated with EEGE were found reduced the GSH levels to half (Figure 4). And this pattern of decrease was seen statistically significant at all concentrations (25, 50, 100 μg/ml, p < 0.01) of EEGE when compared with the control cells.

### Apoptosis induction in EEGE-treated EAT cells

**Figure 5:**
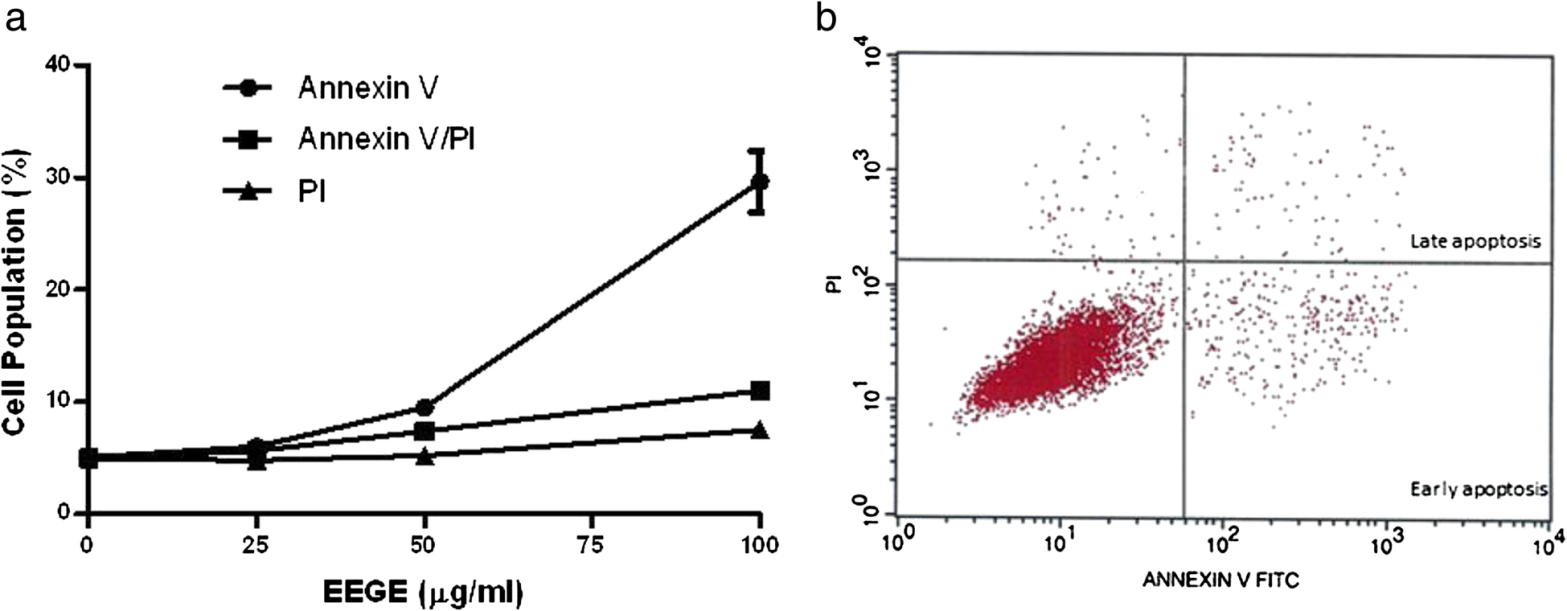
**Effect of EEGE on apoptosis induction.** EAT cells were treated for 72 hours with 25, 50 and 100 μg/ml EEGE for 72 hours and harvested for quantification of Annexin-V-positive, PI-positive, and Annexin-V/PI-positive cells by flow cytometry, **a)** graph indicating increase in the early apoptotic events in EEGE treated cells, **b)** representative dot plot from the flow cytometry data.

**Figure 6:**
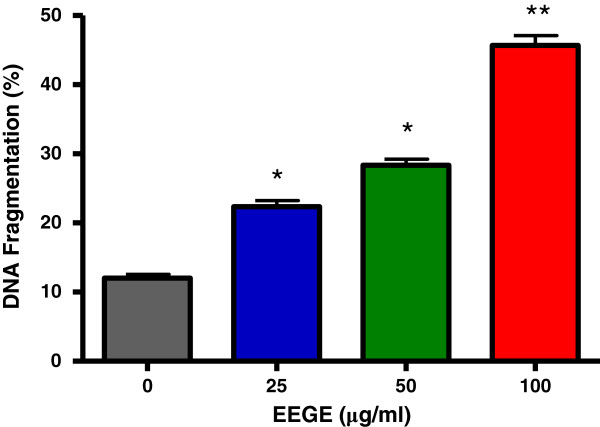
**Percentage data of DNA fragmentation obtained by DPA method in EAT cells (3 × 10**^**5 **^cells/ml) treated with different concentrations of EEGE for 72 hours, *p < 0.01 compared to control cells. **p < 0.01 compared to control cells and to 25 and 50 μg/ml treatments (ANOVA, Tukey test). The results express the mean ± S.D. of three experiments run in duplicate.

**Figure 7:**
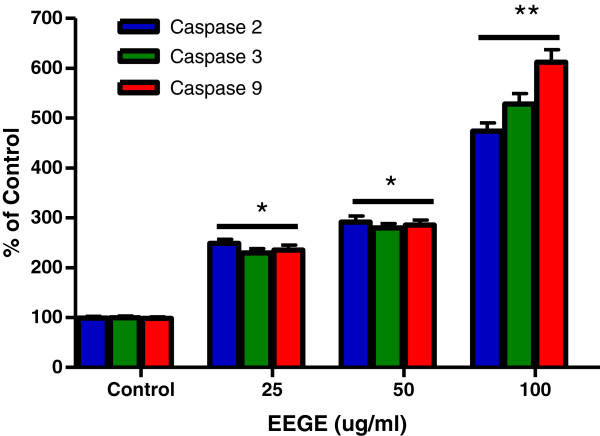
**Alterations on caspase-2, caspase-3 and caspase-9 activities after incubation of EAT cells (3 × 10**^**5 **^cells/ml) with different concentrations of EEGE for 72 hours, *p < 0.05 compared to control cells. **p < 0.01 compared to control cells and to 25 and 50 μg/ml treatment (ANOVA, Tukey test). The results express the mean ± S.D. of two experiments run in triplicate.

To understand the mechanism of cytotoxicity of EEGE to EAT cells, we investigated the nuclear DNA fragmentation based apoptosis approach, a characteristic hallmark of apoptotic cells. As observed in Figure 5, DNA fragmentation in EAT cells was dose-dependently increased with EEGE treatment. The control untreated cells produced 10% of fragmentation, while EAT cells treated with 25, 50, and 100 μg/ml of EEGE for 72 hours produced 21, 27, and 43% of DNA fragmentation, respectively (p < 0.05). These DNA fragmentation observation suggests that EEGE induces EAT cells killing by the process of apoptosis. For detailed understanding of cell death and differentiation between cells undergoing necrosis or apoptosis in the EEGE-mediated cell death, EAT cells were treated with similar concentrations of EEGE as in DNA fragmentation experiment (25, 50 and 100 μg/ml) for 72 hours and analyzed by flow cytometry using PI and FITC conjugated Annexin-V labeling. Changes in membrane phospholipid bilayer, such as externalization of the phosphatidylserine, which can be stained with Annexin-V-FITC, are characteristic of cells undergoing apoptosis. In contrast, loss of membrane integrity, shown by PI staining, has been associated with necrosis. Analysis by flow cytometry of EEGE-treated cells stained with Annexin- V-FITC directed that apoptosis is major factor for cell death as there is significant increases in Annexin-V-FITC positive populations after 72 hours of exposure to 50 μg/ml (p < 0.05) and 100 μg/ml (p < 0.01) EEGE. A considerable increase in Annexin-V-FITC staining of 100 μg/ml over 50 μg/ml treated samples was observed (Figure 5). These results supported the higher DNA fragmentation levels determined in 100 μg/ml EEGE treated cells (Figure 6). In addition, small, but statistically significant (p < 0.05), populations of cells were Annexin-V-FITC/PI double stained after treatment with 50 and 100 μg/ml, while only at the highest dose of EEGE a significant (p < 0.05) PI-positive population could be determined (Figure 5), reflecting cell death by necrosis, which might be related to the longer period of incubation with the algae extract. Significance of caspases in apoptosis very well documented and the role of caspase-2, caspase-3 and caspase-9 in the EEGE induced EAT cell death was examined. After 72 hours of incubation with EEGE, cells treated with 25 μg/ml of the algae extract a significant increase (2 fold) for all caspases activities when compared to the control cells (p < 0.01) (Figure 7). Treatment of cells with 100 μg/ml EEGE resulted in 4.5, 5 and 6-fold increases of caspase-2, caspase-3 and caspase-9 activities, respectively (p < 0.01). These biochemical features, as high DNA fragmentation, low membrane rupture, high phosphatidylserine externalization and activation of effector caspases are most likely indicative of activation of an apoptotic death pathway by EEGE in EAT cells.

### Antitumor evaluation

**Figure 8:**
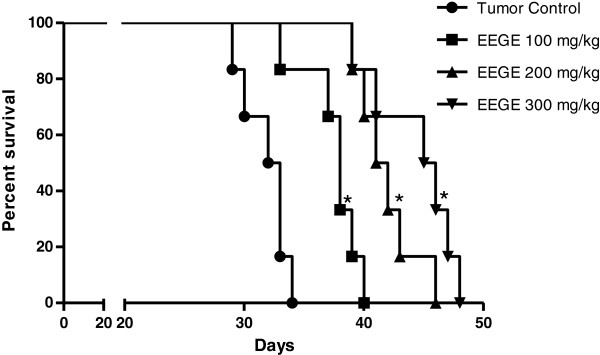
**Survival of EAT cells bearing animals treated with EEGE.** Mice were inoculated i.p. with 6 × 10^6^ cells and after 24 h, they were treated i.p. with daily 0.2 ml injections of 25 μg/ml (♦), 50 μg/ml (▲) and 100 μg/ml EEGE (●) throughout their lifespan. Mice from the control group were treated with 0.2 ml of vehicle, PBS containing 10% Tween 80 (▪). Curves were represented by the method described by Kaplan–Meier and differences among groups were analyzed by Log-rank (Cox–Mantel) test for non-parametric procedures (*p < 0.05 compared with vehicle-treated tumor group and n = 6/group).

With evidence from the *in vitro* studies for the antitumor potential of this algae extract, we continued to investigate with *in vivo* model in this study. The effect of EEGE on the survival time of EAT cells bearing mice was evaluated and is presented in Figure 8. EAT cells were injected intraperitoneally to mice and these cells grew as ascites tumor with accumulation of large volume of ascitic fluid in the peritoneal cavity. Survival of the control group was found to be at 50% on the 32^nd^ day after tumor inoculation and no animal survived beyond the 34^th^ day. Whereas survival of EEGE (300 mg/kg) treated EAT cells bearing animals was 100% on the 38^th^ day and 15% in the 45^th^ day, with no animal alive beyond day 48. All the doses of the algae extract tested in this experiment (100, 200 and 300 mg/kg) significantly altered the rate of mice survival (p < 0.05). No significant statistical difference was observed between mice treated with 100 and 200 mg/kg of EEGE. The administration of 100, 200 and 300 mg/kg of EEGE after tumor inoculation resulted in a significant inhibition of tumor growth (p < 0.05), as evident from a 75% reduction in intraperitoneal tumor cell burden on the day of death. Mice treated with 100, 200 and 300 mg/kg EEGE presented 3.6 ± 2.3 × 10^7^, 3.8 ± 2.1 × 10^7^ and 3.9 ± 2.8 × 10^7^ viable ascites cells, respectively, while the control group presented 12.1 ± 3.4 × 10^7^.

### *In vivo* toxicity studies

**Figure 9:**
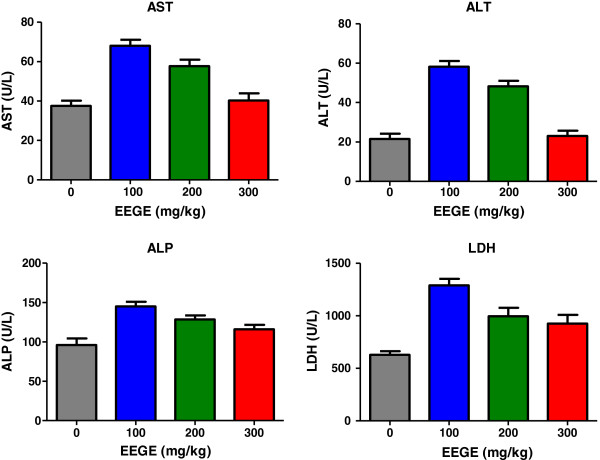
**Aspartate aminotransferase (AST), Alanine aminotransferase (ALT) activities, Alkaline phosphatase, and LDH levels in serum from mice treated with different doses of EEGE.** The results express the mean ± S.D. (n = 6/group). ANOVA was performed for statistical comparison among groups.

**Figure 10:**
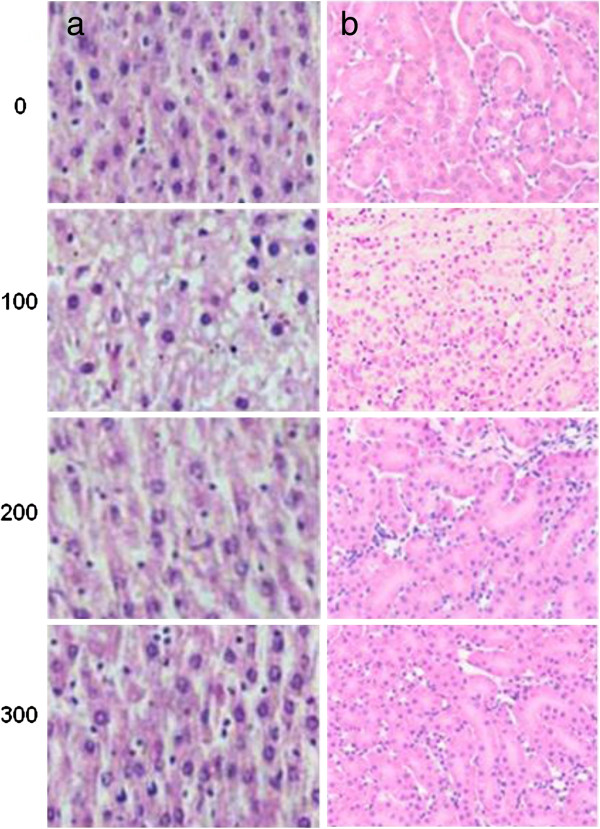
**Histological findings (40×) in liver and kidney of mice treated with different doses of EEGE.** Adult swiss albino mice were treated i.p. with daily 0.2 ml injections of 100, 200 and 300 mg/kg EEGE and on the next day after the last injections, liver and kidney were submitted to routine histological processes. Mice from the control group were treated with 0.2 ml of vehicle, PBS containing 10% Tween 80. **(a)** Normal liver histological findings and **(b)** normal kidney histological findings.

After encouraging effect of EEGE in inhibiting cancer progression *in vivo*, we evaluated the undesired side effects of the i.p. administration of daily doses of 100, 200 and 300 mg/kg of EEGE for 35 days in healthy adult swiss albino mice. Drug toxicity was assessed by clinical signs of gross toxicity, behavioral changes and mortality, including hematological, biochemical and histopathological parameters. No animal death was observed in any of the groups during the experimental period of 35 days. No abnormal clinical signs or behavioral changes were observed in any of the groups, and changes in body weights of the EEGE-treated groups were not significantly different between any groups including the control group after 35-days of treatment period (Table 1). There were no significant changes in hematological parameters in the EEGE-treated groups (Table 2). Similarly, no significant differences were found between the EEGE-treated groups and the controls for the three blood chemical parameters evaluated (Figure 9), AST, ALT, ALP and LDH, which were within the physiological range of values expected for the method of blood collection [27]. These data indicate that daily intraperitoneal injections of EEGE at doses up to 300 mg/kg for 35 days did not cause hematotoxicity nor poses risks of renal or hepatotoxicity. At necropsy, no visible pathological changes were noted in the livers and kidneys of mice administered EEGE at 100, 200 and 300 mg/kg doses. Histological analysis of formaldehyde-fixed, paraffin embedded liver and kidney sections stained with hematoxylin and eosin showed normal architecture in all experimental groups. Livers of animals treated with different doses of EEGE showed no sign of necrosis, fatty degeneration, or inflammation (Figure 10a). Similarly, glomerulus structures, and proximal and distal tubules in kidneys showed normal architecture (Figure 10b), pointing out that EEGE did not cause toxicity to these organs.

**Table 1:** Body weights (g) of control and EEGE treated mice during the period of the study

**Day(s)**	**Control**	**EEGE 100 mg/kg**	**EEGE 200 mg/kg**	**EEGE 300 mg/kg**
	**Mean ± S.D.**	**Mean ± S.D.**	**Mean ± S.D.**	**Mean ± S.D.**
0	23.6 ± 1.18	22.8 ± 0.954	23.4 ± 1.26	22.8 ± 0.954
35	27.1 ± 1.98	27.8 ± 1.33	29.6 ± 2.06	28.3 ± 2.25

**Table 2:** Hematological parameters measured in BALB/c mice treated with different doses of EEGE for 35 days

**Parameter (unit)**	**Control**			**EEGE 100 mg/kg**			**EEGE 200 mg/kg**			**EEGE 300 mg/kg**		
	**Mean ± S.D.**	**Min**	**Max**	**Mean ± S.D.**	**Min**	**Max**	**Mean ± S.D.**	**Min**	**Max**	**Mean ± S.D.**	**Min**	**Max**
RBC (10^6^/μl)	9.44 ± 0.987	8.65	11.83	9.36 ± 1.58	7.68	12.0	8.76 ± 1.12	7.28	10.5	8.90 ± 1.05	8.12	10.8
WBC (10^3^/μl)	5.12 ± 1.56	4.1	6.3	6.21 ± 1.48	4.87	7.23	5.18 ± 1.18	4.78	6.68	4.98 ± 1.47	4.2	6.85
Lymphocytes (%)	79.1 ± 6.34	74.3	88.4	83.2 ± 6.68	77.8	90.5	83.4 ± 11.2	74.9	85.3	79.9 ± 6.37	71.3	83.8
Monocytes (%)	6.4 ± 1.3	5.2	7.8	5.3 ± 1.8	3.52	6.81	5.80 ± 1.67	4.0	8.2	5.3 ± 1.8	4.2	7.5
Eosinophils (%)	1.2 ± 1.1	0	3.2	0.6 ± 0.3	0	1.1	1.4 ± 0.7	0	2.4	1.5 ± 0.70	0.1	2.2
Basophils (%)	0.8 ± 0.2	0	1.4	0.4 ± 0.3	0	1.2	00.9 ± 0.5	0	1.3	0.3 ± 0.15	0	1.1
Platelet (10^5^/μl)	5.64 ± 1.78	3.25	8.21	7.12 ± 2.45	4.13	10.9	6.12 ± 1.98	3.48	9.25	6.45 ± 2.78	3.75	10.2
HGB (g/dl)	13.9 ± 1.58	12.4	17.1	13.4 ± 1.31	11.9	14.9	13.8 ± 1.48	12.1	14.8	13.2 ± 1.45	12.2	14.5
HCT (%)	40.8 ± 3.56	34.8	46.1	39.1 ± 5.11	34.1	47.2	42.1 ± 3.78	35.6	45.8	40.9 ± 4.32	36.2	43.2
MCV (fl)	52.4 ± 2.32	46.21	54.2	48.5 ± 2.36	44.5	51.2	51.3 ± 1.56	46.2	53.8	48.3 ± 1.23	44.8	51.6
MCH (pg)	16.2 ± 1.26	15.2	17.4	17.3 ± 1.49	14.3	18.5	16.4 ± 1.44	15.4	17.4	16.4 ± 1.29	15.4	17.9
MCHC (g/dl)	33.8 ± 3.44	30.4	36.1	32.8 ± 3.49	30.7	35.9	35.8 ± 2.45	31.5	37.9	33.1 ± 2.78	29.4	36.1
PMN (%)	15.6 ± 6.79	15.3	21.35	17.5 ± 5.89	15.56	22.1	17.1 ± 5.81	14.56	21.2	15.8 ± 6.10	13.62	18.99

## Discussion

The nature has been constant source of inspiration and unsolved puzzle as source of medicinal compound [28]; especially the marine source has been a reliable source of novel medicinal molecules [29,30]. The interplay of this source with advanced technologies can be extended towards enhancing the discovery process and it leads to the new hope of investigating new natural products will continue to turn up even useful drugs to treat cancer [31]. In our earlier report [32] we demonstrated an initial investigation of *G. edulis* as a potential candidate for cancer treatment and due its high toxicity in cancer cells. *G. edulis* is a rich source of combination of amino acids, tissue nutrients, and pigments [33], fatty acid, palmitic acid and high protein content [34]. In view of these facts, this study was an attempt to evaluate the *in vivo* and *in vitro* antitumor activity of *G. edulis* against EAT cells and we used the ethanolic extract of the marine product as a known source of pathological activity [35]. In addition, cultures of normal human peripheral blood lymphocytes and a 35-day toxicity study in mice were conducted to determine its possible toxic effects.

The cytotoxicity effect of EEGE was the primary considerations as the significant activity to induce cell death, and this was demonstrated by MTT cytotoxic assay in EAT cells and lymphocytes in addition to phosphatase activity. MTT is reduced to formazan in cells indicating cell redox activity and the reaction is an effect of mitochondrial enzymes and electron carriers [36]. In natural compounds phosphatase activity determination is a successful tool to evaluate cytotoxicity and as a parameter to study the role of the natural compound induced adaptation to apoptosis and oxidative stress [37,38]. The IC_50_ for the compound was similar in these two methods and this cytotoxicity probably reflects the cell response to particular kinds of damage, in this case, mitochondria insult and/or oxidative stress. Additionally the dye exclusion assay using trypan blue also confirmed that the reduction on cell viability and cell number was due to the cytotoxic action of *G. edulis* to EAT cells. In contrast to the activity against the EAT cells, EEGE showed lesser effectiveness in normal human peripheral blood lymphocytes in similar experimental conditions, where the IC_50_ value was about two fold higher than EAT cells. This is in agreement with results previously published for other marine natural products [39]. This reinforces the lower EEGE toxicity for non-tumor cells than for tumor cells and suggesting *G. edulis* as a promising agent for cancer management.

When anticancer agents (whether *in vivo* or *in vitro*) are used for treatment in cancer cell population, large changes may occur in the cell, and in result of that many cells are killed by the treatment, while others remain unaffected, either because they are resistant or because of biochemical, cell cycle, or extra-cellular environmental sanctuaries. The major non-protein thiol of the cell, GSH has chemoprotective action and the ratio of reduced glutathione (GSH): oxidized glutathione (GSSG) is maintained at the optimum by the cell as the ratio is critical to survival; a deficiency of reduced form of glutathione is a risk of cell to oxidative damage since this ubiquitous cellular tripeptide plays a vital role in protecting cells against oxidative damage by free radicals [40]. In many pathological conditions including cancer the ratio is observed as altered [41,42], distinct responses to chemotherapeutic drugs have prompted cellular GSH modulation as target for cancer chemotherapy [43]. Earlier studies have presented evidence of correlation of high GSH content and increased resistance to anticancer agents [44,45]. This could be a cell- or cancer-specific response and would be especially important to find a drug, which can lower the GSH level and helps in sensitivity to certain drugs, radiation and oxygen. GSH level of a cell makes it more resistant to certain antitumor agents, radiation and oxidative effects. On the other hand, therapy that decreases cellular GSH levels usually promotes sensitivity to certain drugs, radiation and oxygen [46]. It is also observed by other investigators GSH plays important roles in antioxidant defense, nutrient metabolism, and regulation of cellular events including gene expression, DNA and protein synthesis, cell proliferation, apoptosis, signal transduction, cytokine production and the immune response [47]. Results obtained in this study indicate that *G. edulis* exhibits a dose-dependent cytotoxicity to EAT cells in parallel with reduced levels of GSH for all concentrations used. Cell death induced by oxidative stress by *G. edulis* may have impact on growth or death related factors and in reduction of intracellular GSH, and conferred altered antioxidant system. From earlier studies reported by numerous investigators it is understood that increase in intracellular ROS and depletion of intracellular GSH to occur with the onset of apoptosis [48-50]. Morphological alterations observed in the EAT cells treated with *G. edulis* such as ruffling, blebbing, condensation of the plasma membrane, and the aggregation of nuclear chromatin were in concurrent with the initial hypothesis. Involvement of ROS production in colon HT29 cells death induced by natural products derived from marine source demonstrated by individual investigators [51,52] and the nuclear fragmentation investigated as proof of induced apoptosis in oral squamous cell carcinoma cells [53]. Current drugs commonly used in anticancer therapy induce apoptosis in target cells, and it involves both receptor-mediated and mitochondrial-mediated pathways. Disruption of the mitochondrial membrane potential, cytochrome c release and activation of different caspases have already been described following treatment of EAT cells with different natural agents [54,55]. DNA fragmentation is observed as an initial event in apoptosis and is considered to occur at an early stage of apoptosis [56]. In this present study we observed that apoptosis was an associated event in EAT cells after incubation with *G. edulis* and increase in the percentage of fragmented DNA quantified by the diphenylamine method, which occurs concurrently with an increase in Annexin-V-FITC positive cells as an indicator of apoptosis. EAT cells incubated with *G. edulis* demonstrated increase in all caspase evaluated including caspase-2, caspase-3 and caspase-9. Caspases are important members in apoptosis mediated cell death and it is well-known that the ROS level may influence the membrane potential in mitochondria, and these caspases in mitochondria induce release of pro-apoptotic factors by caspase cascade activation [57]. Upstream caspases in mitochondria are activated by pro-apoptotic signals from the cytoplasm leads to proteolytic activation of downstream caspases like caspase-3, followed by cleavage of set of vital proteins and apoptotic degradation phase is initiated in the cell including DNA degradation and the typical morphologic features [58]. Cell death by apoptosis was also reported in glioma cell after treatment with marine sponge, which was correlated with the elevation of ROS and calcium levels, the impairment of mitochondrial function and the activation of caspases [59] and DNA degradation [60]. Depletion of glutathione is reported to be associated with apoptosis following enhanced cell death of tumor cells where the essential sulfhydryl group of glutathione is lost resulting in a changed calcium homeostasis and ultimately loss of cell viability. ROS generation by *G. edulis* treatment in the EAT cells leads to increase in reduced GSH contents and may contribute to the cell death.

The results from this study indicate *G. edulis* exhibited antiproliferative and apoptotic activities against EAT cells *in vitro* and is a promising candidate for extensive screening in animal model. To the best of our knowledge, we report the *in vivo* antitumoral activity of *G. edulis* for the first time. An expected rapid increase in ascites tumor volume was observed in EAT cells bearing mice. Ascitic fluid serves as direct nutritional source for tumor cells and is highly essential for tumor growth and a rapid increase in ascitic fluid meets the nutritional requirement of tumor cells [61]. Animals treated with low doses of *G. edulis* inhibited the tumor volume, viable tumor cells count and increase survival rate of EAT cells bearing mice, opposed to the reports with high doses of compound from natural products from variety of sources including marine. Even though the mechanism of action by which *G. edulis* is able to produce these significant results is still not clear, observed properties like changes in ROS production, GSH level and activation of apoptosis followed by cell death may be the contributing factors towards its anticancer activity. Mice bearing EAT cells showed increase in survival time after treatment with *G. edulis* deserves further investigation. This is first kind of study exploring the pharmacological activities especially the anti-tumor activities of *G. edulis* and consistent toxicity study of *G. edulis in vivo*, where the complete hematology is described, and the liver and kidney functions were investigated by biochemical determination of AST, ALT, ALP and LDH levels and histopathological examination of these tissues in mice given daily i.p well tolerated doses of 100, 200, 300 mg/kg of EEGE. Animals treated with *G. edulis* showed no clinical signs of gross toxicity or change in behavior. And the treatment did not affect the body weight gain in comparison with the control group.

## Conclusion

Results from this study from all experiments congregate to a noticeable observation of the antitumoral activity of *G. edulis* on EAT cells *in vitro* and *in vivo*, and there was no considerable toxicity to major organs in mouse model. It is important to mention that cautious observation of such natural products from marine source to be a significant candidate in antitumor and apoptosis inducing drug group and to combat human cancer where formation of peritoneal malignant ascites is a fundamental basis of morbidity and mortality.

## Abbreviations

EEGE: Ethanolic extract of *Gracilaria edulis*; EAT: Ehrlich ascites tumor; ROS: Reactive oxygen species; i.p.: Intraperitoneally; FBS: Fetal bovine serum; HEPES: 4-(2-hydroxyethyl)- 1-piperazineethanesulfonic acid; DMSO: Dimethyl sulfoxide; MTT: 3-(4,5-dimethiazol-zyl)-2-5-diphenyltetrazolium bromide; GSH: Reduced glutathione; DTNB: 5-5′′-Dithio-bis(2-nitrobenzoic acid); pNA: p-nitroanilide; GSSG: Oxidized glutathione; DCFH: Dichlorofluorescein; DCFH-DA: 2′, 7′-Dichlorodihydrofluorescin diacetate; FITC: Fluorescein isothiocyanate; PI: Propidium iodide; AST: Aspartate aminotransferase; ALT: Alanine aminotransferase; ALP: Alkaline phosphatase.

## Competing interests

The authors declare that there are no conflicts of interests.

## Authors’ contributions

SP designed the experiments and wrote the manuscript; SP and MMS participated in the experiments; MMS provided algae material and contributed to the manuscript writing process. Both authors read and approved the final manuscript.

## Pre-publication history

The pre-publication history for this paper can be accessed here:

http://www.biomedcentral.com/1472-6882/13/331/prepub
